# Comments on the need for a new look at public policies on food and nutrition in early childhood

**DOI:** 10.1590/0102-311XEN121223

**Published:** 2023-10-23

**Authors:** Gisele Ane Bortolini

**Affiliations:** 1 Ministério do Desenvolvimento e Assistência Social, Família e Combate à Fome, Brasília, Brasil.; 2 Ministério da Saúde, Brasília, Brasil.

A new look at social policies must combine structuring actions with integrated measures focusing on the synergistic determinants of all forms of malnutrition and guarantee access to public policies that can contribute to whole child development. These actions must also ensure equity and social participation; reduce regional, racial, and gender inequalities; and consider the recent increase in food and nutritional insecurity in Brazil since 2022 estimates suggest that 125 million Brazilians suffered from some degree of food and nutritional insecurity, most prevalently in the Brazilian North and Northeast, female-headed households with children, and homes with self-reported black or mixed-race guardians ^1^.

Castro et al. ^2^ described nutritional transition in Brazilian children < 5 years old from 2006 to 2019, marking important advances and challenges to be considered in a new cycle of public policies on food and nutrition in early childhood. The period showed considerable increases in maternal schooling, water and internet access, and basic sanitation, a lower anemia and hypovitaminosis A prevalence, a greater overweight rate, and a short stature and breastfeeding stable trend ^2^.

The Brazilian scenario of anemia and hypovitaminosis, outlined by ENANI-2019, improved national policies for micronutrient supplementation, which are no longer universal and have begun to focus on those who need it most: all children living in the North Region and children registered in the Brazilian Unified Registry for Social Programs and the beneficiaries of the Brazilian Income Transfer Program in other regions of the country ^3^. It is now necessary to guarantee access to micronutrientes by strengthening implementation and monitoring and integrating it with other health actions and public policies that guarantee the human right to adequate and healthy food for all Brazilians.

Short stature (an indicator that also measures the development of a country) showed no improvement in the evaluated period. In the preceding decade (1996 to 2006), Brazil showed an about 50% lower prevalence of short stature (13.5% to 6.8%), two-thirds of this reduction were attributed to the favorable evolution of the four studied factors: maternal schooling, purchasing power, health care, and health conditions ^4^. Malnutrition negatively impacts children’s health and the development of a country by reinforcing the intergenerational transmission of poverty and other inequalities. Despite the improved social and demographic indicators from 2006 to 2019, short stature remained the same, evincing the need for the resumption of policies that guarantee income, education, access to health, basic sanitation, and the rescue of the main food and nutrition security public policies that helped Brazil leave the Hunger Map in 2014.

Of the 6.3 million children < 5 years old evaluated by the primary health care of the Brazilian Unified National Health System (SUS) in 2022, 6.04% (n = 380,993) showed underweight; 5.34% (n = 337,883), short stature; 6.35% (n = 400,943), obesity; 7.9% (n = 498,544), overweight, and 17.82%, a risk for overweight (n = 1,125,099) ^5^. Health, food, and nutrition surveillance data not only support the organization of health care for each family in a given basic health unit or community, but also guide actions in other sectors. Children the health sector finds to show malnutrition, for example, must have a therapeutic plan built for them, have ensured access to social assistance services, and be a priority in food and nutritional security actions so they can receive food from the Brazilian Food Acquisition Program in the case that lack of food has determined their malnutrition. Intersectoral integration must happen across territories to effectively guarantee rights, social protection, and care, especially of the most vulnerable populations.

Breastfeeding indicators increased but without statistical differences between the studied periods. The actions the World Health Organization (WHO) recommend are already recognized worldwide and include actions to promote, protect, and support breastfeeding, especially by guaranteeing maternal and paternity leaves. In Brazil, only public servants’ maternal leaves are consistent with the recommendations for exclusive breastfeeding up to 6 months according to the *Dietary Guidelines for Brazilian Children Under 2 Years of Age* ^6^. However, most Brazilian women enjoy neither the protection of this right nor adequate or sufficient leaves. Such model fails to support exclusive breastfeeding during children’s first 6 months of life and reinforces gender inequalities by delegating the act of caring to mothers most of the time. A change to this reality would require national congress bills that guarantee this right to all Brazilian families.

It was observed the high consumption of ultra-processed foods alarming as it referred to almost 90% of all evaluated children. The increasingly early consumption of ultra-processed foods impairs the formation of healthy eating habits and negatively impacts children’s health, influencing weight gain and overweight and configuring an urgent need for public policies that protect children from the consumption of ultra-processed foods.

Excess weight increased in the period, raising an alert for the need for public policies for integrated prevention and promotion in the first years of life. Excess childhood weight increases the chance of children remaining overweight during early school age, adolescence, and adulthood. The Brazilian National School Feeding Program, which guarantees healthy meals for more than 40 million students, recently had its recommendations aligned with those of the Brazilian food guides, prohibiting ultra-processed foods for children < 2 years old and restricting ultra-processed foods for those of other ages ^7^. Preventing childhood obesity includes guaranteeing structuring, health, and intersectoral actions ^8^. Moreover, schools are an important space to promote, protect, and support adequate and healthy food.

Primary health care interventions from the SUS that have been indicated as effective and recommended for all forms of malnutrition include the monitoring of gestational weight gain; guidance on adequate and healthy eating, breastfeeding, and healthy complementary feeding in children’s first years of life, and surveillance of infants’ growth ^9^. A longitudinal study conducted in Brazil with children in the Brazilian Income Transfer Program showed that those who primary health care routinely followed for four years or more had lower overweight and malnutrition prevalence rates ^10^. However, induction mechanisms must be adopted to routinely perform effective food and nutrition actions and guarantee this actions for all children and pregnant women cared in a basic health unit.

The prevention of all forms of malnutrition requires a discussion on the need for a healthier and more sustainable food system and for food guides to drive policies in all sectors. Different sector policies can stimulate the production of healthy food needs and prioritize the production of agroecological foods and family farming. Such healthy food must reach families and be close to people’s homes, thus requiring a national supply policy that guarantees access and fiscal policies that ensure that healthy foods have affordable prices and that ultra-processed food costs discourage their consumption. School and work environments must be regulated to only offer healthy foods; the advertising of ultra-processed foods to children, prohibited; and the Brazilian norm of food trade for infants and children in early childhood, implemented.

Promoting and protecting children’s health requires a proactive and articulated action by the Brazilian State, formulating structuring and converging policies and actions that enable children a full life and offer them the possibility of reaching their highest development potential with health, well-being, and a good quality of life.
